# Flash Graphene-Modified Portland Cement Paste with Both High Electromagnetic and Mechanical Performances at a Low Percolation Threshold

**DOI:** 10.3390/ma19020266

**Published:** 2026-01-08

**Authors:** Zixiao Wang, Zhen Zhang, Wenqing Shen, Weizheng Shi, Tingting Liu, Wenyu Li, Aming Xie

**Affiliations:** 1 School of Safety Science and Engineering, Nanjing University of Science and Technology, Nanjing 210094, China; 2 School of Mechanical Engineering, Nanjing University of Science and Technology, Nanjing 210094, China; 3 State Key Laboratory of Silicate Materials for Architectures, Wuhan University of Technology, Wuhan 430070, China

**Keywords:** flash graphene, percolation threshold, electromagnetic wave absorption, cement paste, portlandite

## Abstract

The contradiction between the threshold values of carbon nanomaterials in cement-based materials for enhancing electrical, magnetic, and mechanical properties appears irreconcilable in previous studies. Reducing the numerical differences of these thresholds of carbon nanomaterials in cement-based materials is a straightforward approach to resolving the predicament. Flash graphene powder (FGP) with varying dosages is used to prepare the modified Portland cement paste in this work. Hydration heat release behaviours and the morphologies of hydrates are significantly impacted due to the unique turbostratic graphene layers. The percolation threshold of FGP in paste approximates its thresholds for enhancing the strength and absorption of electromagnetic waves (EMWs), which is 0.50 wt.% of cement. The compressive and flexural strength values of samples with 0.50 wt.% FGP increased by 59.5% and 22.4%, respectively, compared with the blank sample. The minimum EMWs loss value of the sample with 0.50 wt.% FGP is −12.2 dB with an effective absorption bandwidth value of 7.76 GHz in the EMWs frequency between 2 and 18 GHz. The smaller Portlandite crystals are associated with better conductive and impedance-matching properties, resulting in significantly improved EMWs absorption in the Ku band. This work proposes a possible solution that involves using FGP to replace normal graphene, thereby alleviating the contradiction and reducing the gaps in the graphene thresholds in cement paste and enhancing mechanical and electrical conductivity and EMWs absorption properties.

## 1. Introduction

Public concern over electromagnetic pollution and signal leakage has been growing with the rapid development of modern information technology and the widespread application of wireless communication systems, as well as electronic and electrical equipment [1]. The popularisation of 5G networks, Internet of Things devices, radar systems, and high-frequency data transmission technologies has significantly increased the density of electromagnetic waves (EMWs) in the built environment, leading to growing concerns about potential interference between electronic systems and their adverse effects on human health and equipment performance. Therefore, effectively preventing and mitigating electromagnetic pollution and ensuring the information security of important facilities have become a focus for interdisciplinary experts [2,3,4,5,6]. The demand for materials that can absorb rather than reflect EMWs is also increasing to reduce secondary pollution while lowering detection risks in stealth applications. Cement-based materials are among the most widely used building materials in modern construction projects, attributed to their excellent mechanical properties, structural designability, and good durability.

However, in the absence of any functional phases, the DC resistivity of cement paste is about 1000 kΩ·cm, indicating that cement paste is an insulator [3,7]. Cement-based materials are not conducive to supporting eddy current losses or dielectric polarisation mechanisms, which are crucial for the effective absorption of EMWs [8,9]. Additionally, ordinary cement-based materials, due to their non-magnetic and extremely low dielectric loss characteristics, have a relatively low ability to dissipate electromagnetic energy [10]. Therefore, for incident EMWs, cement-based materials largely utilise wave interference, similar to the design of polarising films, dissipating their energy through quarter-wavelength interference cancellation. However, a notable feature of materials with this absorption mechanism is the narrow absorption bandwidth of EMWs frequencies. Especially when the wave impedance at the interface of cement-based materials does not match that of free space, there may be no effective EMWs absorption bandwidth.

Due to their good resistance to oxidation in high-alkaline environments, carbon-based nanomaterials, such as graphene [11,12,13,14], carbon nanotubes [15,16,17], carbon fibres [17,18,19], and carbon nanospheres [17], are the most studied as conductive materials for adjusting the impedance-matching properties of cement-based materials. However, according to the results in the literature, when carbon-based nanomaterials exceed a certain dosage, a decline in the mechanical properties of cement-based materials is inevitable [1,20,21], including brittleness and a decrease in compressive strength. According to the literature [21], the percolation threshold of graphite nanoplatelets in cement paste is between 2 and 3 wt.% of cement, at which the dosage of graphite nanoplatelets sharply weakens the compressive strength of the paste. A more detailed description is as follows: if the mechanical performance of cement-based materials is required to be high, the electromagnetic performance will decline; conversely, if the electromagnetic performance is required to be good, the mechanical performance will be poor. Therefore, this significantly restricts the promotion and application of EMWs-absorbing cement-based materials. The contradiction in cement-based EMWs-absorbing materials remains a challenging point and an urgent problem to be addressed in current research.

Joule heating flash evaporation technology utilises the resistance of the sample itself to achieve high temperatures (>3000 K) and cooling of the sample in seconds [22,23,24], offering significant advantages in the microstructural regulation and large-scale production of carbon materials. Flash graphene [25,26] is a new type of high-purity graphene material prepared in a very short time (at the millisecond level) using Joule heating flash evaporation technology. Its raw materials are widely available [27,28,29], including carbon black, coal tar pitch, petroleum coke, straw, wood chips, tyre rubber, and other carbon-based solid wastes. The reparation cost of the flash graphene is significantly lower than that of graphene prepared by other methods. Flash graphene is partially composed of turbostratic graphene layers that have rotational misalignment between them, and the rest are rippled graphene layers. The main component of graphene treated by common thermal annealing methods is rippled graphene. When a current directly acts on carbon-based materials, high-quality turbostratic flash graphene can be formed, which is a new feature and performance enhancement introduced to graphene materials by Joule heating flash technology. Turbostratic graphene can be easily peeled off by shearing without requiring large amounts of solvents or high-energy mechanical shearing for peeling, indicating that the surface properties of flash graphene materials will bring new changes to multiphase interface performance.

Cement hydrates are formed by the hydration of cement particles in water, whose morphologies and contents are sensitive to the patterns and microstructures of the added graphene particles. Considering the unique turbostratic morphology of the flashed graphene, introducing the flashed graphene particle to cementitious material seems a possible strategy for releasing the mismatches among the percolation threshold, the optimal dosage for enhancing strength and EMWs-absorbing properties. Since its introduction in 2020, flash graphene has received extensive attention from scholars worldwide. Some researchers have added flash graphene to cement-based materials [28,30,31,32,33], with results showing that adding a small dosage of flash graphene can enhance compressive strength, electrical conductivity, and rheological properties. However, previous literature has not focused on the correlation between mechanical and electromagnetic properties in flash graphene-modified cement-based materials. Barely any research has focused on the EMWs-absorbing performance change of flash graphene-modified cement-based materials. The contradictory phenomena mentioned above between strength and electrical–magnetic properties require further study.

In this study, the relationships involved in the enhancement of the strength, electrical, and magnetic performance of flash graphene on cement paste samples are investigated. Typical flash graphene powders, produced from carbon black powders, are used to make the tested samples. The effects of flash graphene particles on the hydration of Portland cement are examined by hydration heat analysis. Chemical phase analyses are conducted to describe the changes in the main hydrates caused by the different dosages of flash graphene at 3-day age and 28-day age. Physical properties, including thermal conductivities and porosity, are also detected. Additionally, the relationship between the flash graphene electrical percolation threshold and the strength enhancement thresholds in cement-based paste is discussed in detail. The relationships among the EMWs’ absorption performance, conductivities, and the variation of Portlandite are also addressed.

## 2. Experimental Section

### 2.1. Materials and Sample Preparation

**Figure 1:**
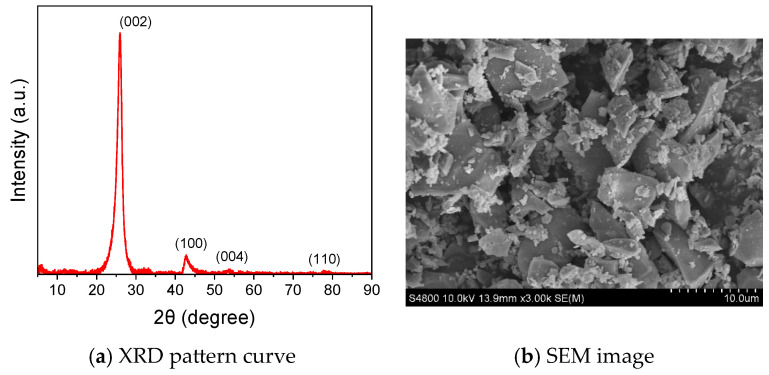
XRD pattern curve and SEM image of flash graphene powders.

The P.I. 42.5 Portland cement powders and the commercial flash graphene powders (FGP) were used to prepare cement paste samples. The chemical composition of the cement powders, listed in Table 1, was measured by an X-ray fluorescence spectrometer (Axios, Malvern Panalytical Ltd., Malvern, UK). The flash graphene powders were produced from carbon black powders by the flash Joule heating process, provided by Taiyuan Saiyin New Materials Co., Ltd. (Taiyuan, China). The XRD pattern curve and an SEM image of the FGP are shown in Figure 1. An asymmetric (002) peak, as shown in Figure 1a, appeared along with a weak (100) peak, which is typically displayed in turbostratic graphene [34]. The particle sizes of the FGP ranged from 2 to 10 µm, as shown in Figure 1b. The electrical conductivity value of FGP was approximately 3.4 S/mm, measured by a four-probe resistance tester (FT-300, Ningbo Ruike Micro Intelligence Technology Co., Ltd., Ningbo, China) under a test pressure of 10 MPa. The BET specific surface area value of the FGP was 167 m^2^/g, measured by a specific surface area and porosity analyser (TriStar II Plus, Micromeritics Instrument Corporation, Norcross, GA, USA).

The mass ratios of FGP to cement in the paste samples in each group were 0, 0.25 wt.%, 0.50 wt.%, 0.75 wt.%, and 1.0 wt.%. The water-to-cement ratio was 0.4. The appropriate amounts of hydroxypropyl methylcellulose (HPMC) powders were added to the slurries to adjust the viscosity of the fresh paste samples. Firstly, the FGP was dispersed in the mixing water using a magnetic stirrer for 30 min, at a stirring speed of between 1500 and 2500 rpm. After the cement and HPMC powders were mixed evenly in the mixer, mixing water containing FGP was added to the powders, and the mixture was stirred for an additional 3 min. The slurry was cast into samples of two different sizes (200 × 200 × 20 mm^3^ and 40 × 40 × 160 mm^3^) for different tests. All samples were demoulded after 24 h and then cured in a curing chamber at a temperature of 20 ± 2 °C and a relative humidity higher than 95% until the test age.

### 2.2. Characterisations

#### 2.2.1. Basic Characterisation

An X-ray diffractor (XRD, SmartLab9, Rigaku, Tokyo, Japan) was used to investigate the mineralogical phases of powder samples at 1-day age and 28-day age. The test parameters of the X-ray diffractor were set as a period time of 0.5 s, an increment of 0.02, scanning 2θ degree ranges from 5° to 90°, and a testing voltage of 40 kV by a copper target. Before the XRD tests, the samples were crushed into fine powder with a particle size of less than 75 µm. According to previous work [35], the crystals of Portlandite (CH) have a hexagonal prism shape faceted by {10–10} prismatic and {0001} basal facets, and the aspect ratio (r_L_) of the crystal can be calculated by

(1) $$r_{L}=L_{1}/L_{2}$$

where L_1_ and L_2_ are the linear sizes of Portlandite crystal in the {10–10} and {0001} growth direction, respectively.

The microstructures of the paste samples at 1 day and 28 days of age in each group were recorded by a scanning electron microscope (S-4800 II, Hitachi, Tokyo, Japan) with an accelerating voltage of 20 kV. The samples, with a size of less than 10 mm^3^, were processed and spray-coated with a layer of platinum, then vacuum-dried before the test. An automatic mercury porosimeter (AutoPore V 9600, Micromeritics, Norcross, GA, USA) was used to measure the pore size distribution curves of the samples at 1 day and 28 days of age. Before the test, the hardened samples were cut into small pieces, approximately 2 to 4 mm in size. In this work, the measured pore sizes of the samples were divided into three classes: mesopores with a pore size between 2 and 50 nm, macropores with a pore size between 50 and 7500 nm, and megapores with a pore size larger than 7500 nm [36].

#### 2.2.2. Cement Hydration Heat

An isothermal calorimeter (TAM Air, TA Instruments, New Castle, DE, USA) was used to record the heat release curves of cement hydration with different dosages of FGP at 10 s intervals for the first 72 h of hydration. The procedure involved sealing approximately 15 g of fresh slurry in a test container. The test container was then placed in the test chamber, and a separate container of deionised water was placed in the corresponding test channel as a reference sample. The test temperature was 20 ± 0.001 °C.

#### 2.2.3. Thermal Decomposition of FGP-Modified Paste

Thermogravimetry curve analyses of paste samples of different ages were conducted using a thermal analyser (TGA/DSC3+, Mettler-Toledo, Greifensee, Switzerland) with a heating rate of 10 K/min from 50 °C to 1000 °C and the carrier gas was pure nitrogen. Before the TGA test, the samples were crushed into fine powders with a particle size of less than 75 µm.

For Portland cement-based materials, the physically bound water evaporates between 30 °C and 105 °C. The gypsum, AFt, and AFm phases dehydrate at 110 °C to 170 °C. C-S-H gels begin to dehydrate at around 100 °C and decompose between 120 °C and 320 °C. Portlandite decomposes at temperatures between 450 °C and 550 °C.

#### 2.2.4. Thermal Conductivity of FGP-Modified Paste

**Figure 2:**
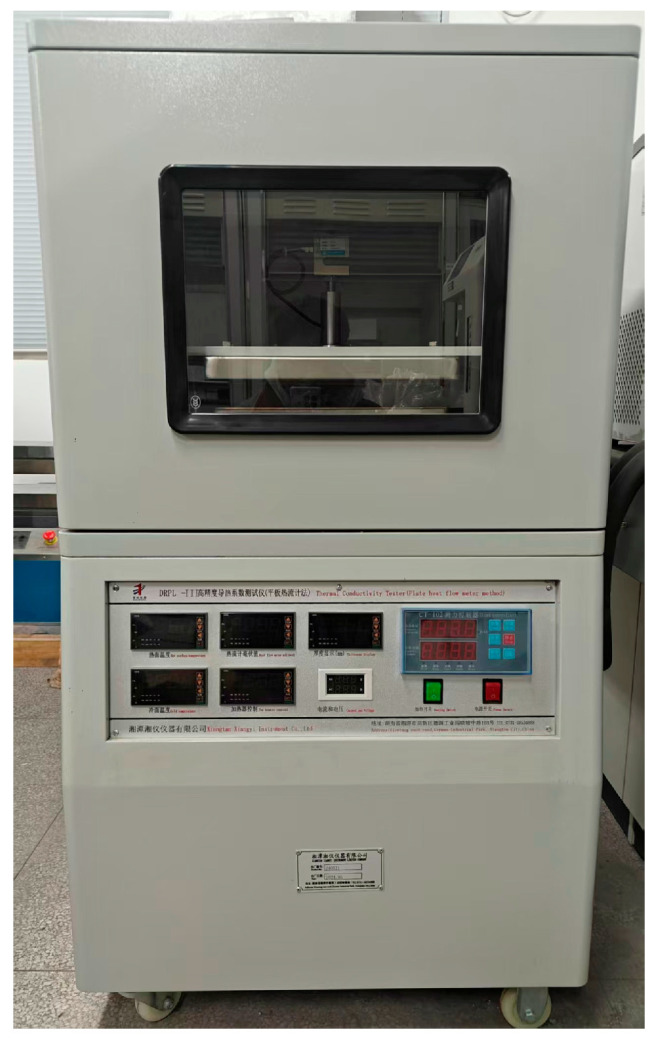
Photo of the thermal conductivity tester.

As shown in Figure 2, a thermal conductivity tester (DRPL-III, Xiangtan Xiangyi Instrument Co., Ltd., Xiangtan, China) was used to measure the thermal conductivity values of paste samples with a size of 200 mm × 200 mm × 20 mm. The measurement accuracy of the tester is 0.10 mW/(m·K). A low-constant-temperature water bath with a temperature control accuracy of 0.01 °C was also used during the test. The ambient temperature was maintained at 25 ± 2 °C, and the relative humidity was 65 ± 5 % RH during the test.

#### 2.2.5. Electrical Resistivity of FGP-Modified Paste During Curing

**Figure 3:**
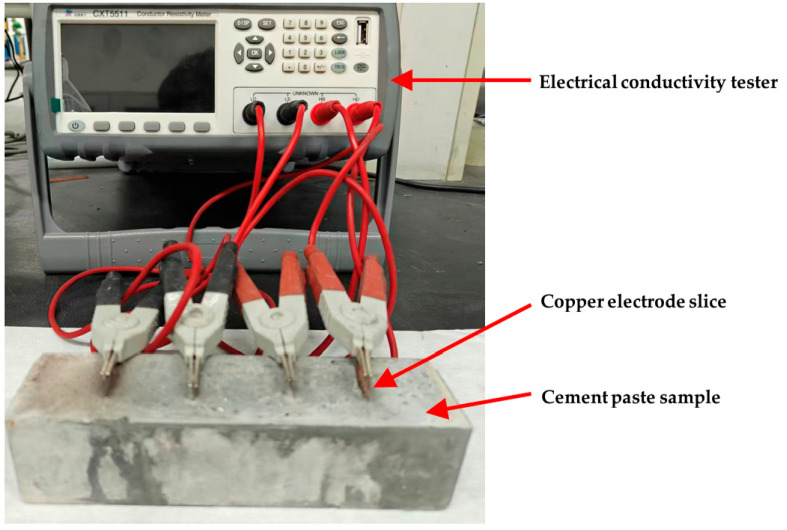
The electrical conductivity tester and the prism samples with four electrode slices.

Electrical resistivity is a typical parameter used for estimating the electrical conductivity ability of cementitious materials. In this study, the four-electrode direct current (DC) electrical resistivity values of paste samples at the ages of 1, 2, 3, 4, 5, 6, 7, 14, 21, and 28 days were measured by a resistivity tester (CXT5511, Changzhou Xinyang Electronic Technology Co., Ltd., Changzhou, China) at ambient temperature. As shown in Figure 3, three parallel cement paste samples in each group with a size of 40 × 40 × 160 mm^3^ were used for electrical resistivity tests. Four copper slices (20 × 50 × 0.38 mm^3^) were embedded in the prism samples as electrodes. The copper electrodes were set at 32 mm, and the outer electrodes were placed 32 mm from the edge of the prism samples. The highest resistance accuracy of the tester was 0.02%, the minimum resolution of resistance was 0.1 µΩ, and the test range was from 0.1 µΩ to 10 MΩ. During the test, the ambient temperature was about 25 ± 1 °C, and the relative humidity was about 65 ± 5%.

#### 2.2.6. Flexural and Compressive Strength of FGP-Modified Paste

The flexural and compressive strength values of the paste samples were determined using a universal testing machine (WAW-1000, Shenzhen SUNS Technology Stock Co., Ltd., Shenzhen, China) with a loading rate of 2.4 kN/s. Three parallel prism-shaped samples with a size of 40 × 40 × 160 mm^3^ of each group were used for the flexural strength measurement. Six half-prism samples from the flexural strength measurement were used for the uniaxial compression testing.

#### 2.2.7. EMWs Reflection Loss (RL) of FGP-Modified Paste

**Figure 4:**
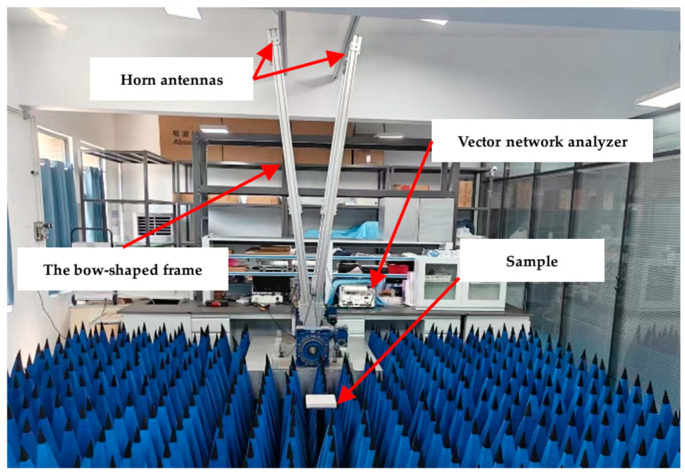
The test system of the EMWs reflection loss of slab-like samples via the bow-frame method.

A vector network analyser (N5222B, Keysight Technologies, Santa Rosa, CA, USA) was used to test the EMWs absorption performance of cement paste slab-like samples (200 × 200 × 20 mm^3^) from 2 GHz to 18 GHz using the bow-frame method [11,37,38]. The measuring system for RL curves is shown in Figure 4. All samples were dried at 55 °C for 48 h to remove free water, minimising the influence of water content on the test results [18,39]. The vector network analyser was calibrated before each test. The recorded data represents the average values of three tests for each sample. The system was intended to measure the reflected power from a reflective aluminium plate (P_m_) and the sample (P_a_). The EMWs reflection loss (RL) of the test sample was calculated using Equation (2) [18]:

(2) $$RL=10\mathrm{lg}\frac{P_{m}}{P_{a}}$$

The EMWs absorption characteristics are studied through the measurement of RL curves. Based on the reflection principle [13,40], more than 80% and 90% of the EMWs energy could be translated into thermal energy in the test samples when the RL was lower than −7 dB and −10 dB, respectively. In this study, the frequency range where the RL value is below −10 dB was designated as the effective absorption bandwidth (B_e_) for the samples.

## 3. Results

### 3.1. Effects of Flash Graphene on Cement Hydration

#### 3.1.1. Hydration Kinetic

**Figure 5:**
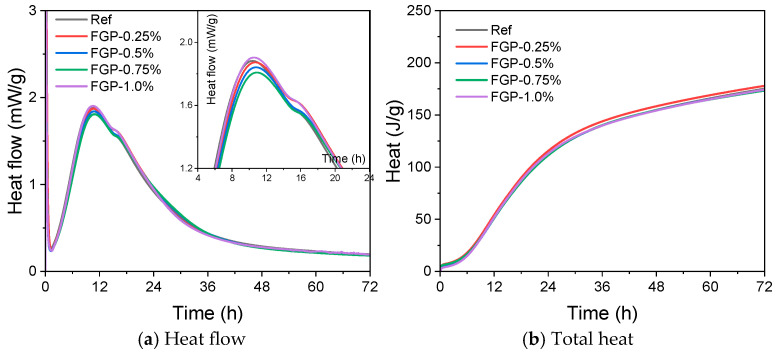
Influences of FGP on the exothermic heat flow and total heat per gram of cement.

Figure 5 shows the heat flow and total heat curves of cement paste slurries containing different dosages of FGP during the first 72 h of hydration. In Figure 5a, the peak values of the second hydration heat evolution peaks initially decreased and then increased with the dosage of FGP in the slurry, indicating that the formation and precipitation of calcium hydroxide crystals during the acceleration period of Portland cement were significantly influenced. However, during the deceleration periods, the hydration of C_3_S was also influenced by the presence of FGP with different dosages; all of the values of the third peak in the heat flow curves increased with the FGP dosages.

Flash graphene is partly composed of turbostratic graphite layers with rotational misalignment between them, and the remainder consists of crumpled graphene sheets, which resemble amorphous carbon [25,34]. Therefore, the above-mentioned phenomena may be attributed to the variation in the nuclear effects of flash graphene micron particles, along with the graphene content in the cement slurries of each group. According to Figure 5b, the differences between the total heat curves of each group were small, meaning the influences on cement hydrates at a longer age were negligible.

#### 3.1.2. Hydration Products

**Figure 6:**
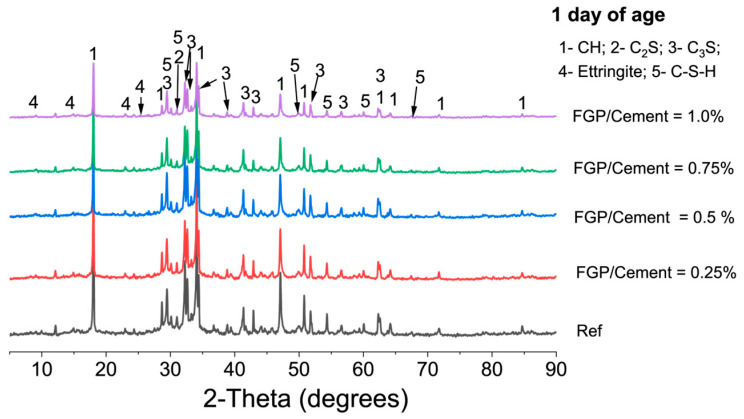
X-ray diffractograms of paste containing different dosages of FGP at 1 day of age.

**Figure 7:**
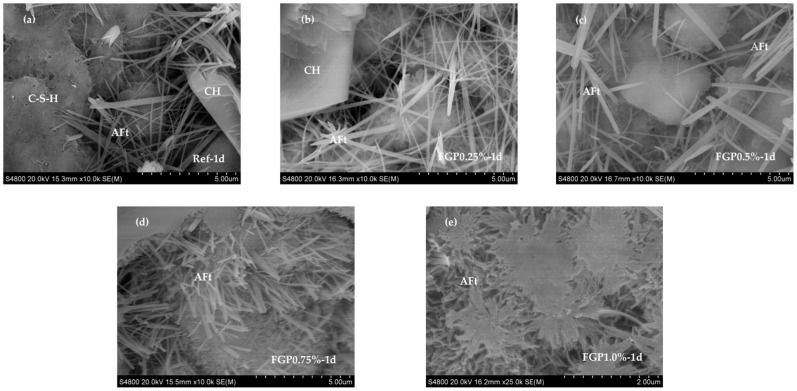
Morphology of Ettringite in the cement paste at 1 day of age with different dosages of FGP: (**a**) 0 wt.% FGP; (**b**) 0.25 wt.% FGP; (**c**) 0.5 wt.% FGP; (**d**) 0.75 wt.% FGP; (**e**) 1.0 wt.% FGP.

Figure 6 shows the XRD spectra of hardened cement paste containing different dosages of FGP at 1 day of age. The presence of FGP does not affect the patterns of cement hydrates, which include Ettringite, Portlandite, and C-S-H phases. However, the morphologies of Ettringite crystals at an early age are significantly influenced. The peak values at 9.1 degrees and 15.8 degrees have weakened with the increase in FGP dosage, indicating that the needle-shaped crystals become shorter and thinner. According to the SEM images shown in Figure 7, the detected Ettringite crystals in the blank sample without FGP are typical hexagonal rod-shaped crystals. With the increase in FGP-to-cement mass ratios in the hardened paste sample, the Ettringite crystals become shorter and thinner. In particular, in the hardened paste sample containing 1.0 wt.% FGP, the Ettringite crystals are clusters with blurred edges, as shown in Figure 7e.

**Figure 8:**
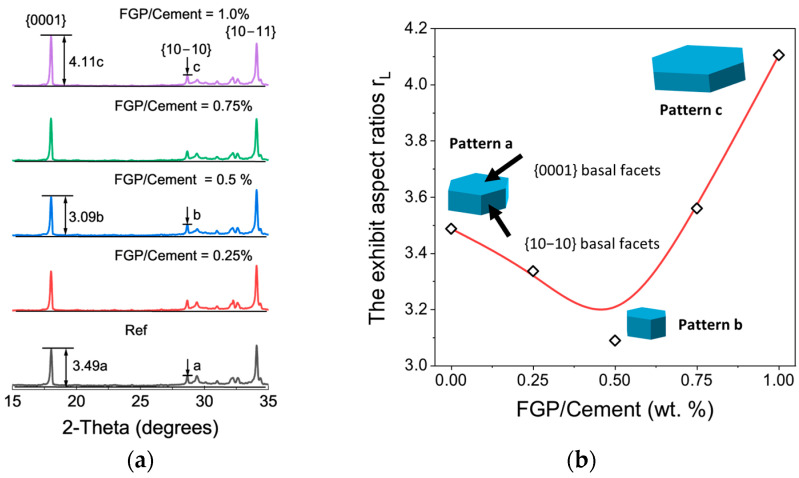
(**a**) Crystal pattern of Portlandite of cement paste at 28 days of age; (**b**) plot of the aspect ratios (r_L_) of Portlandite vs. FGP mass content.

Figure 8a shows the XRD pattern of paste samples containing different contents of FGP at 28 days of age. The typical peaks of Portlandite at {0001}, {10–10}, and {10–11} planes appear on the patterns of all samples, indicating that the presence of FGP does not affect the lattice parameters of Portlandite crystals. However, the intensities of facets of {0001} and {10–10} change slightly with the dosages of FGP in the paste samples. In Figure 8b, the aspect ratios (r_L_) of Portlandite crystals first decrease and then increase with the dosages of FGP in the paste samples, which corresponds with the crystalline shape change in Portlandite [41].

**Figure 9:**
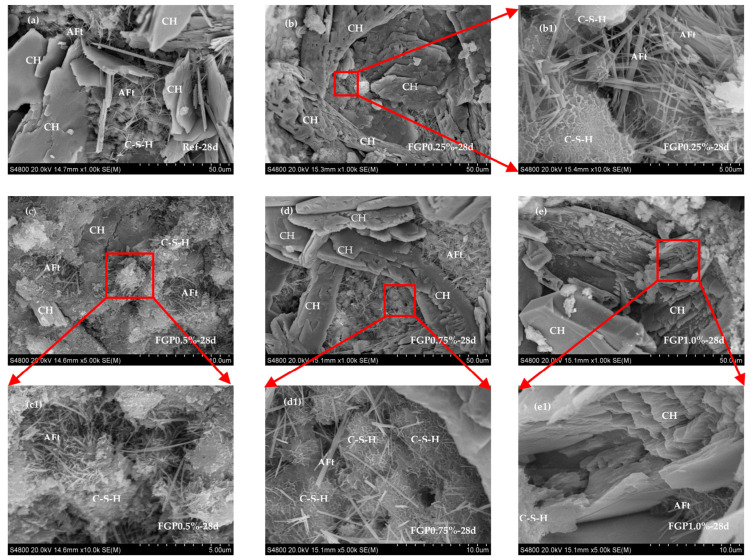
Morphology of Portlandite in the cement paste at 28 days of age with different dosages of FGP: (**a**) 0 wt.% FGP; (**b**) 0.25 wt.% FGP; (**c**) 0.5 wt.% FGP; (**d**) 0.75 wt.% FGP; (**e**) 1.0 wt.% FGP; (**b1**–**e1**) are the images of the detection areas marked with red boxes in (**b**–**e**) at a higher magnification.

Overall, the Portlandite crystals exhibit a hexagonal plate-like morphology, as illustrated in pattern a of Figure 8b. With decreasing r_L_ values, the crystals elongate along the c-axis and display more clearly defined lateral crystal faces (see pattern b of Figure 8b). In contrast, higher r_L_ values result in shorter crystals along the c-axis and more pronounced {0001} basal faces (as shown in pattern c of Figure 8b). As depicted in Figure 8a, CH crystals are typically hexagonal and plate-shaped, characterised by a relatively small {0001} basal face area and well-defined, regular edges. Furthermore, as shown in Figure 9a–c, in hardened paste samples containing less than 0.5 wt.% FGP relative to cement, CH crystals tend to exhibit smaller and more layered growth at 28 days, accompanied by an increase in both the number and area of {10–10} prism faces. These phenomena may be due to the influence of the dosages of FGP particles on the nucleation effects of cement hydrates. Therefore, the mass contents of the main hydrates in the hardened paste samples with different dosages of FGP may differ, resulting in varying performances on the macro scale.

**Figure 10:**
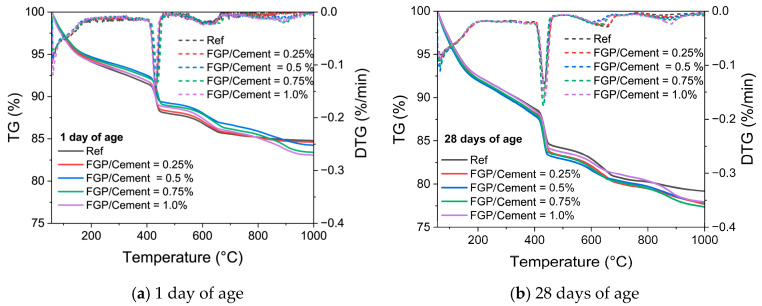
TG and DTG curves of paste with different dosages of FGP at 1 day and 28 days of age.

Figure 10 presents the thermogravimetry curves of the paste sample at 1 day and 28 days of age. In Figure 10, the temperature ranges of thermal decomposition of the main hydrates are relatively stable. The TG curves also support the notion that the presence of FGP in the paste samples does not affect the patterns of the main hydrates, but rather the mass contents of the hydrates. Table 2 lists the mass contents of Portlandite, C-S-H, and non-evaporable water in paste samples containing different dosages of FGP at 1 day and 28 days of age. According to the data in Table 2, at 1 day of age, the CH contents in paste samples first increase and then decrease with the increase in FGP-to-cement mass ratios. In contrast, the non-evaporable water and C-S-H contents exhibit an opposite changing law, which first decreases and then increases with the FGP-to-cement mass ratios in the samples. At 28 days, the contents of CH and non-evaporable water in the paste sample containing FGP are larger than those in the reference sample, while the contents of C-S-H in each tested group are close to each other.

### 3.2. Porosity and Strength

**Figure 11:**
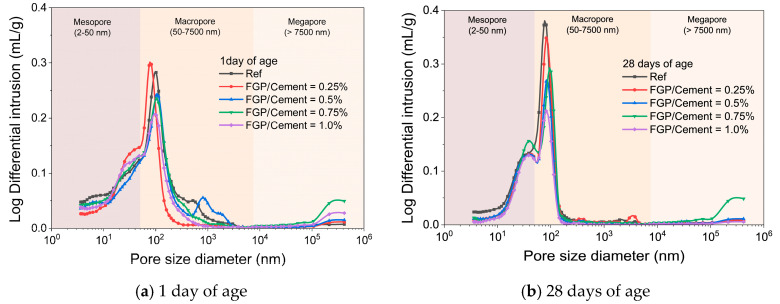
The pore size distribution curves of paste with different dosages of FGP at 1 day and 28 days of age.

**Figure 12:**
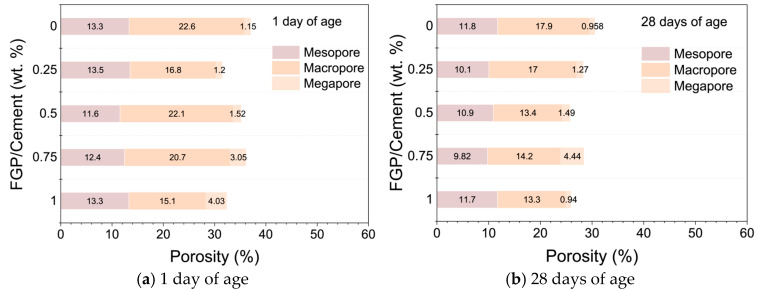
The porosity of the paste with different dosages of FGP at 1 day and 28 days of age.

Figure 11 shows the pore size distribution curves of samples containing different dosages of FGP measured by a mercury porosimeter at 1 day and 28 days. Figure 12 shows the measured porosity values for different pore sizes in each sample group. In Figure 11, it is easy to see that there are three peaks in each curve of the samples at 1 day of age. The first peak is in the range of 2 to 50 nm (mesopore), the second and the third peaks are in the range of 50 to 7500 nm (macropore), and the third peak is in the range of above 7500 nm (megapore). At 28 days, there are only three peaks in each curve; the first peak is in the range of 2 to 50 nm, the second peak is in the range of 50 to 100 nm, and the third peak is in the range of above 7500 nm. The values of the first and second peaks in pore size distribution curves at 28 days of age decrease obviously in the samples containing FGP. In Figure 12a, at 1 day of age, the porosity of mesopores first decreases and then increases slightly with the increase in the FGP-to-cement mass ratios, which may be because of the morphological variation of hydrates (AFt phases), as mentioned in Section 3.1.2. At 28 days, the sum of porosity of mesopores and macropores shows a similar changing law, which also first decreases and then increases slightly with the increase in the FGP-to-cement mass ratios. According to the classical Power’s porosity–strength theory [42], the strength increases with the decrease in capillary voids (smaller than 50 nm). Therefore, the strength values of the hardened paste samples initially increase and then decrease with the increase in the FGP-to-cement mass ratios. The following section will discuss the test data for the compressive and flexural strength values of samples at various ages.

**Figure 13:**
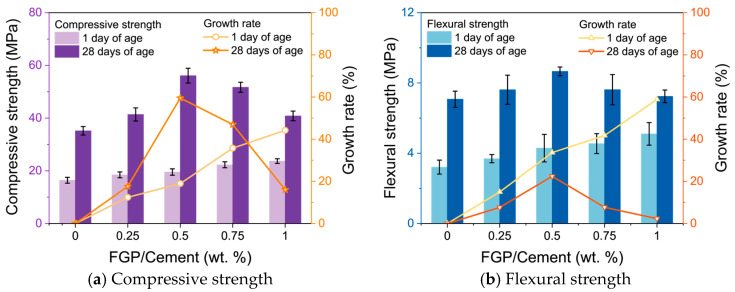
Compressive and flexural strength values of paste with different dosages of FGP at 1 day and 28 days of age.

Figure 13 shows the compressive and flexural strength values and their growth rates of the hardened cement paste containing different dosages of FGP at 1 day and 28 days of age. At an early age, the compressive and flexural strength values of samples containing FGP increase with the mass ratios of FGP to cement. At 28 days, the sample with a mass ratio of FGP to cement of 0.5 wt.% obtains the highest compressive and flexural strength values, with growth rates of 59.5% and 22.4%, respectively. As mentioned in Section 3.1.2, the Portlandite in the hardened paste samples grows in layered crystals with smaller shapes when the FGP-to-cement mass ratio is lower than 0.5 wt.%, and grows into larger crystals with indistinct boundary edges of layers when the FGP-to-cement mass ratio exceeds 0.5 wt.%. The strength results prove that the morphologies of the main hydrates affect the strength values of the FGP-modified cement paste samples. Smaller Portlandite crystals result in higher compressive and flexural strengths of the hardened paste.

### 3.3. Thermal and Electrical Conductivities

**Figure 14:**
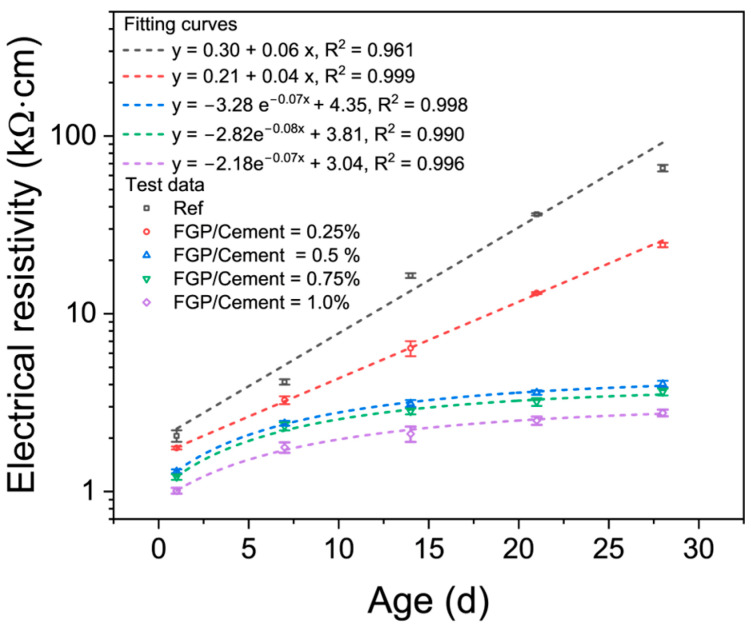
Electrical conductivity of paste with different dosages of FGP during curing.

**Figure 15:**
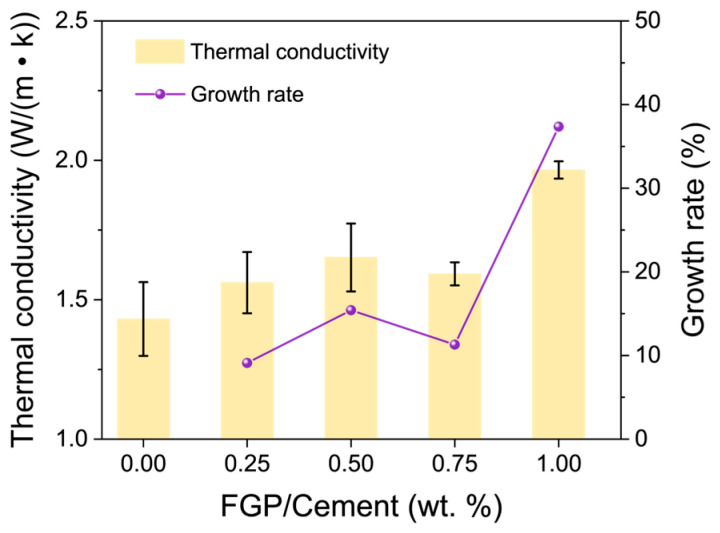
Thermal conductivity of paste with different dosages of FGP at 28 days of age.

As a carbon material, FGP exhibits high electrical and thermal conductivity values. Figure 14 shows the electrical conductivity values of the hardened paste samples with varying FGP dosages during the curing process. In Figure 14, the symbols represent the test data, and the dashed lines represent the fitting curves. Figure 15 shows the thermal conductivity values of cement paste samples at 28 days of age.

As shown in Figure 14, when the FGP-to-cement mass ratio is smaller than 0.25%, the electrical conductivity values of the paste samples increase linearly with the curing age, and the slope values of the fitting lines exhibit an opposite changing law. When the FGP-to-cement mass ratios are larger than 0.50%, the electrical conductivity values of the paste samples increase exponentially with curing age, which tends to stabilise when the curing age exceeds 21 days. At 1 day of age, the electrical conductivity values of the paste samples in each group are relatively small, but the electrical conductivity values decrease with the increase in FGP dosages in the samples, with the lowest value being about 1.0 kΩ·cm of the sample containing 1.0 wt.% of FGP. After 14 days of curing, the difference in electrical conductivity values of samples in each group becomes significant. At 14 days of age, the electrical conductivity value of the sample in the reference group is about 16.4 kΩ·cm, while the value of samples containing FGP is still smaller than 10 kΩ·cm, especially the value of samples with FGP-to-cement mass ratios larger than 0.50 wt.%, where the electrical conductivity values are smaller than 5 kΩ·cm. At 28 days of age, the electrical conductivity value of samples in the reference group is about 66 kΩ·cm, and the value of samples with 0.25 wt.% of FGP is about 24 kΩ·cm. In contrast, the electrical conductivity values of the samples are smaller than 5 kΩ·cm when the FGP-to-cement mass ratios are larger than 0.50 wt.%. The reduction rates of the electrical conductivity values of the sample containing FGP are 63.1%, 93.3%, 94.5%, and 95.8%, when the FGP-to-cement mass ratios are 0.5%, 0.75%, and 1.0%, respectively.

Figure 15 shows the change in the thermal conductivity values of the paste samples with the FGP-to-cement mass ratios at 28 days of age. It is easy to see that the thermal conductivity values of the hardened paste samples increase with the increase in FGP dosages. When the FGP-to-cement mass ratios exceed 0.50 wt.%, the growth rates of thermal conductivity values increase by more than 10%, representing a significant rise. The thermal conductivity value of the sample with 1.0 wt.% FGP is approximately 2.0 W/(m·K), which is about 38% lower than that of the reference paste sample. Larger thermal conductivity values of paste samples indicate more transmission and release of cement hydration heat in the matrix, leading to a more uniform temperature distribution and reduced thermal stress within the cement composites.

### 3.4. EMWs Reflection Loss

**Figure 16:**
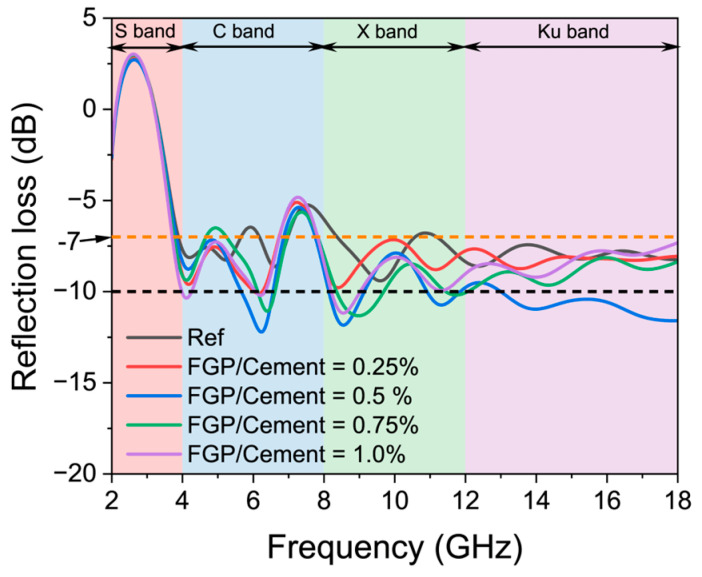
EMWs reflection loss curves of paste with different dosages of FGP at 28 days of age.

Figure 16 presents the EMWs reflection loss curves of the hardened paste samples with different dosages of FGP at 28 days. Table 3 lists the effective absorption bandwidth (B_e_) values of the samples at 28 days. In Figure 16, it is evident that all of the reflectance loss values of samples in the reference group are larger than −10 dB, indicating that more than 10% of the electromagnetic energy is reflected. Thus, the B_e_ of the samples in the reference group is 0, as shown in Figure 16. As seen in Figure 16, there are six typical troughs in each curve, with the values shifting to smaller frequencies as the amount of FGP dosage increases. In the vertical coordinate direction of Figure 16, the values of all curves first move downward and then upward as the FGP dosage increases.

According to the data in Figure 16 and Table 3, the B_e_ values of the FGP-modified cement paste samples in the ranges of X and Ku bands are larger than those in the S and C bands, which may be because of the poor magnetic loss ability of the cement-based materials and carbon-based materials. However, in the C band, the B_e_ values of samples with an FGP-to-cement mass ratio of 0.50 wt.% are the largest, which is approximately 0.96 GHz. The B_e_ values of samples in the C, X, and Ku bands increase first and then decrease with the increase in FGP-to-cement mass ratios. In the high-frequency bands, for example, in the Ku band, the B_e_ value of the sample containing 0.50 wt.% of FGP is about 5.00 GHz, meaning that more than 90% of the EMWs energy in the frequency range of 13 to 18 GHz is effectively absorbed in the sample. Moreover, the total B_e_ value in the frequency range of 2 to 18 GHz for the sample containing 0.50 wt.% of FGP is 7.76 GHz, which is the highest among all tested groups. As a result, the presence of a proper dosage of FGP in cement paste can improve the impedance-matching property of the composite matrix.

## 4. Discussion

As mentioned in the previous sections, the mechanical and functional performances of FGP-modified cement paste samples are closely related to the dosages of FGP and the morphologies of the main hydrates. In particular, the compressive and flexural strength values, as well as the EMWs effective absorption bandwidth values, of the hardened samples follow a similar changing rule with the dosages of FGP in the samples. Therefore, establishing potential relationships among the properties, the main hydrates, and the FGP dosages is beneficial for a comprehensive understanding of the enhancing effects of FGP on Portland cement-based materials. The electrical percolation threshold of FGP in Portland cement-based materials, as well as its differences from other carbon-based materials, will be discussed in this section.

### 4.1. Electrical Percolation Threshold and Strength Enhancement Thresholds

**Figure 17:**
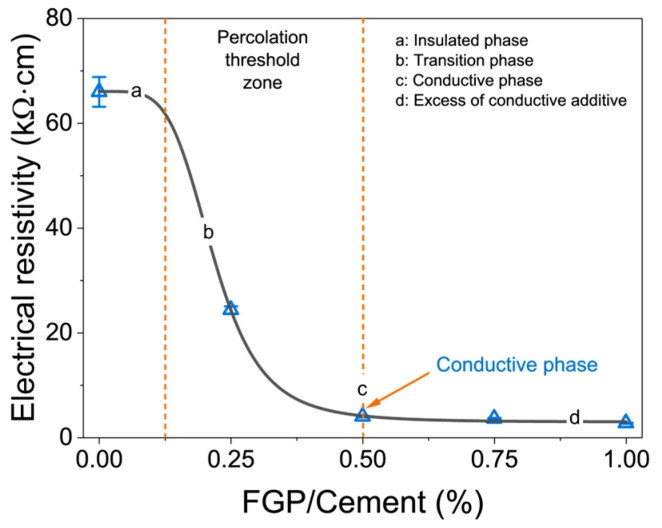
Change in electrical resistivity along with the FGP dosages at 28 days of age.

Figure 17 shows the changing curve of the electrical conductivity of the hardened cement paste and the FGP contents in the samples. According to the general concepts [43,44], a typical conductive cement-based material can be divided into four different phases based on the contents of conductive materials, as shown in Figure 17: the insulated phase (segment a of the curve), the transition phase (segment b of the curve), the conductive phase (segment c of the curve), and the excess phase of conductive additive (segment d of the curve). The conductive phase of the cement-based material refers to the maximum content of the conductive materials in the matrix. In Figure 17, the electrical percolation threshold of FGP is 0.5 wt.% of cement, indicating that the electrical conductivity of the modified cement paste has reached its maximum value at a mass ratio of 0.5% between FGP and cement.

Table 4 lists the electrical percolation thresholds of graphene-modified Portland cement-based materials in the literature and in this work. The thresholds of compressive strength enhancement of graphene on cement-based materials are also listed. Here, the threshold of compressive strength enhancement refers to the maximum dosage of graphene in cement-based materials that yields the largest compressive strength values. In general, these two thresholds of graphene in cement-based materials are quite different. The electrical percolation threshold of one kind of graphene in cement-based material is larger than the threshold of compressive strength enhancement. The threshold of compressive strength enhancement of graphene will not be studied if the topic focuses on the percolation threshold of graphene in cement-based materials and vice versa.

Due to the influence of water-to-binder ratios on the percolation thresholds of conductive fillers in cement-based materials, a water-to-binder ratio of approximately 0.4 to 0.5 is used for better comparability between the results from different studies, as shown in Table 3. For the commonly used graphene listed in Table 4, such as graphite nanoplatelets, multi-layer graphene, and exfoliated graphite, the percolation thresholds are larger than 1 wt.% in cement. In comparison, the thresholds of compressive strength enhancement of these conductive materials on hardened cementitious samples are significantly smaller than their percolation thresholds by one order of magnitude [45,46]. This may be the main reason why most studies only focus on either the mechanical enhancement or the electrical enhancement of graphene on cement-based materials. As listed in Table 4, the percolation thresholds of graphene materials in cement-based materials have been fully studied in the cited literature, whereas the enhancement of graphene on the mechanical properties of hardened cement-based materials has been less mentioned.

**Table 4:** Electrical percolation thresholds of graphene-modified Portland cement-based materials in this work and in the literature.

Conductive Materials	Type	Binder	W/B	Percolation Threshold (wt.% to Binder)	Threshold of Compressive Strength Enhancement (wt.% to Cement)	Ref
GNP	paste	cement + silica fume	0.4	2 to 3	<1	[21]
GNP	mortar	cement	0.485	10	not studied	[47]
MLG	paste	paste + silica fume	0.5	2.5	not studied	[48]
MLG	mortar	cement + fly ash + silica fume	0.375	>0.75	0.5	[49]
EG	mortar	cement	0.4	2	<1	[50]
EG	mortar	cement	0.5	0.8	<0.4	[51]
FGP	mortar	cement	0.4	not studied	0.04	[32]
FGP	mortar	cement	0.45	not studied	not studied	[28]
FGP	paste	paste	0.4	0.5	0.5	This work

GNP: graphite nanoplatelet; MLG: multi-layer graphene; EG: exfoliated graphite; FGP: flash graphene powder. The test method for electrical resistivity is the direct current method.

According to the above discussion, the mismatch between the percolation threshold of flash graphene and the maximum dosage required for strength enhancement has been eliminated in this work. As mentioned in Section 3.1, the presence of flash graphene powder significantly affects the morphology of Portlandite crystals, rather than their content in hardened cement-based materials. It appears that, as one of the primary cement hydrates, Portlandite crystals play a significant role in adjusting both the electrical and mechanical properties of the hardened cement paste. The following section will discuss the numerical relationships between the aspect ratios of Portlandite and properties in cement-based materials at 28 days of age.

### 4.2. Relationships Among EMWs Absorption Performance, Conductivities, and Other Properties

**Figure 18:**
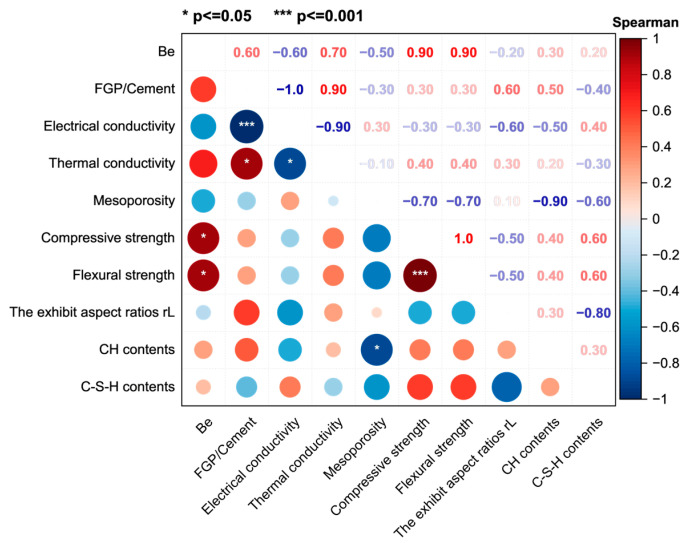
Relationships among B_e_ and the FGP dosages, porosity, conductivity, mechanical strength, main hydrate contents, and CH aspect ratios of the hardened paste samples.

Figure 18 shows the Spearman rank correlation matrix for the B_e_, FGP-to-cement mass ratios, conductivities, porosity, and strength values of the hardened cement paste samples at 28 days of age. The Spearman correlation matrix represents a non-parametric statistical measure that evaluates the strength and direction of a monotonic relationship between two variables, such as those represented by the horizontal and vertical axes. The correlation coefficient ranges from −1 to +1. A positive coefficient value means a positive association, while a negative value means a negative association, and a value of 0 implies no association between the two variables. The absolute values of Spearman rank coefficients between 0.4 and 0.6 are moderate, while those between 0 and 0.3 are weak [26,27] and are generally considered too weak to be considered a significant result. The asterisks in the lower triangular grids in Figure 18 are the *p*-values in the statistical analysis, representing the significance level of parameters on the horizontal axis to those on the vertical axis. One asterisk, two asterisks, and three asterisks indicate that the *p*-values are smaller than 0.05, smaller than 0.01, and smaller than 0.001, respectively. For example, the significance levels of the change in electrical conductivity and the FGP/cement ratios are much higher than those of the compressive and flexural strength values of the samples.

In Figure 18, the correlation coefficient between B_e_ and compressive strength is 0.9, indicating that the B_e_ value increases strongly with the increase in the compressive strength value. The value of the correlation coefficient between B_e_ and r_L_ is smaller than −0.3, meaning a weak negative rank correlation. In other words, this result suggests that the change in the crystal shape of Portlandite (r_L_) does not affect the variation of B_e_ in the hardened cement paste sample. Here, it is worth mentioning that the correlation coefficients between B_e_, electrical conductivity value, and FGP-to-cement mass ratio are 0.6, indicating a positive monotonic relationship between B_e_ and the other parameters. The coefficients between the electrical conductivity of the paste sample and the FGP-to-cement mass ratio and the coefficients between the electrical conductivity and r_L_ are 0.6, indicating the same correlation significance between electrical conductivity and these two parameters. The coefficient between B_e_ and mesoporosity is −0.5, meaning that the monotonic relation between them is not significant. This result suggests that the impedance-matching performance induced by air volumes in the FGP-modified cementitious samples is not the dominant factor contributing to the improvement in EMWs absorption.

Since both the electrical conductivity properties and the dielectric loss of typical cement-based materials are poor [52], adding conductive fillers into cement-based materials is a commonly used method to improve the EMWs-absorbing performance of the hardened matrix [1]. The electrical conductivity of the cement-based material contributes to the imaginary part of the dielectric constant of the EMWs-absorbing cement-based material, which corresponds to the ability to dissipate electric energy [53,54]. When the imaginary part of the dielectric constant of a composite material cannot be ignored, the material acquires the ability to absorb electromagnetic energy. Thus, the EMWs absorption abilities of the conductive cement-based materials are supposed to be closely related to their electrical conductivities rather than other factors. However, based on the results in Figure 18, there is a moderate monotonic relation between B_e_ and the electrical conductivity of the hardened cement paste containing FGP in this work. Moreover, the absolute value of Spearman rank coefficients between B_e_ and the strengths (compressive and flexural strength) is much greater than that between B_e_ and the electrical conductivity of the paste samples, indicating stronger positive monotonically changing relationships between B_e_ and the strength values of the samples. Here, the value of the Spearman rank coefficient between strength and C-S-H contents is 0.6, and the Spearman rank coefficient between C-S-H contents and r_L_ is −0.8. Thus, it can be speculated that both B_e_ and the strength values of the sample increase with a decrease in r_L_. In other words, the smaller crystal shape of Portlandite suggests better EMWs absorption and the higher strength of the FGP-modified cement paste.

Based on the above discussion, the change in the crystal shape of Portlandite in the FGP-modified hardened cement paste sample affects the connection paths between cement hydrates, leading to a change in the impedance-matching property and the electrically conductive paths of the cementitious sample. Therefore, the enhancement rules induced by the presence of FGP on the strength property of the hardened cement paste closely correspond to those on the EMWs absorption property. In this work, the FGP dosage thresholds for strength enhancement, electrical conductivity, and EMWs absorption property correspond to a value of 0.5 wt.% of the cement.

## 5. Conclusions

This work investigates the effects of a new type of graphene, flash graphene (FGP), on the electromagnetic wave absorption and mechanical properties of fresh and hardened Portland cement paste samples. Due to the different influences of FGP on the main hydrates, the modified cement paste samples show the best strength enhancement and the best electrical conductivity and electromagnetic wave absorption at the same threshold of flash graphene. The following conclusions can be drawn:

(1) The aspect ratios of Portlandite crystals at 28 days of age and the formation rates at an early age vary with the dosages of FGP powder in cement paste. Due to the special morphology of the FGP, which is composed of the turbostratic graphite layers with rotational misalignment and the crumpled graphene sheets, the cement hydration during the acceleration period is accelerated in the presence of FGP. The aspect ratios of Portlandite crystals first decrease and then increase with the increase in FGP dosages in the hardened cement paste at 28 days of age. The compressive and flexural strength values of the modified cement paste samples at 28 days of age first increase and then decrease with the dosages of FGP. When the FGP-to-cement ratio is approximately 0.50 wt.%, the compressive and flexural strength values of the samples are the highest in the tested groups, with growth rates of 59.5% and 22.4%, respectively.
(2) The percolation threshold of flash graphene powders in cement paste approximates its threshold for the enhancement of compressive strength. The thermal conductivity values of FGP-modified cement paste increase with the dosage of FGP. The dosages of FGP in paste samples directly impact the aspect ratios of Portlandite crystals. When the FGP-to-cement ratio is about 0.50 wt.%, the Portlandite obtains the minimum value, meaning a much smaller size of calcium hydroxide crystals, indicating a weaker hindrance effect on the transfer of electrons in the hardened cement paste. The maximum percolation threshold of FGP in cement paste with a water-to-binder ratio of 0.4 in this work is approximately 0.50 wt.% of the cement mass, which is a significantly smaller threshold value than that of other types of graphene.
(3) Both the electromagnetic loss capacity and the impedance-matching property of FGP-modified Portland cement paste are improved with a proper dosage. The adjustment of the impedance-matching properties of cementitious matrix with Portlandite of smaller crystalline sizes is much stronger than that induced by the variation in air volume (referred to as porosity). The smaller Portlandite crystals not only enhance the conductivity of FGP-modified cement paste, but also enhance its impedance-matching property, resulting in significantly better EMWs absorption in the wave frequency range of 2 to 18 GHz. The effective absorption bandwidth of the paste sample with 0.50 wt.% of FGP is approximately 7.76 GHz, encompassing the entire Ku band frequency range. This work provides a possible solution to the contradictory relationship between the enhancement of electrical and strength properties when using normal graphene as the conductive and electromagnetic wave-absorbing phase in Portland cement paste, where FGP particles replace normal graphene.

The above conclusions are based on the investigation results in this study. However, more studies related to the long-term properties and EMWs absorption performance of samples in other wave frequency bands should be investigated in future work. Moreover, the EMWs absorption properties of the flashed graphene-modified cement paste samples in this work could be optimized. The proper structural design of the matrix, for example, introducing more air into the hardened matrix, would be a possible way to adjust the impedance matching between the outside air and the cementitious matrix. Further efforts are needed to assess the applicability of the influence mechanisms of cement hydrates on regulating the thresholds of other nanocarbon materials in cementitious materials.

## Figures and Tables

**Table 1:** Chemical composition of Porland cement (wt.%).

Components	CaO	SiO_2_	Al_2_O_3_	MgO	Fe_2_O_3_	SO_3_	K_2_O	Na_2_O	TiO_2_	P_2_O_5_	Minor Elements ^a^
Content	59.761	20.561	5.023	4.613	3.973	3.324	1.159	0.702	0.346	0.147	0.391

^a^ Cr_2_O_3_ + MnO_2_ + CuO + ZnO + SrO + Cl.

**Table 2:** Ca(OH)_2_, non-evaporable water and C-S-H contents of cement paste.

FGP/Cement (wt.%)	CH (%)		Non-Evaporable Water (%)		C-S-H (%)	
	1 d	28 d	1 d	28 d	1 d	28 d
0	18.23	24.04	9.85	13.35	27.15	37.47
0.25	18.93	26.56	9.73	14.10	25.31	38.42
0.50	19.56	26.09	9.04	14.03	22.36	37.67
0.75	20.92	27.04	9.33	14.05	24.11	37.53
1.00	20.16	26.35	9.11	13.79	23.23	36.99

**Table 3:** Effective absorption bandwidth (B_e_) (dB) of cement paste with FGP at 28 days of age.

	B_e_	S Band	C Band	X Band	Ku Band	Total
FGP/Cement						
0		0.00	0.00	0.00	0.00	0.00
0.25%		0.00	0.20	0.00	0.00	0.20
0.5%		0.00	0.96	2.04	5.00	7.76
0.75%		0.00	0.46	1.86	0.06	2.38
1.0%		0.24	0.24	0.80	0.00	1.28

## Data Availability

The original contributions presented in this study are included in the article. Further inquiries can be directed to the corresponding authors.
